# Cultivation Model of Entrepreneurship From the Perspective of Artificial Intelligence Ethics

**DOI:** 10.3389/fpsyg.2022.885376

**Published:** 2022-06-29

**Authors:** Chunzheng Yang, Cuixia Lin, Xiaohuan Fan

**Affiliations:** ^1^Moral Culture Research Center of Hunan Normal University, Changsha, China; ^2^School of Music and Dance, Hunan First Normal University, Changsha, China

**Keywords:** entrepreneurship, independent entrepreneurship, cultivation, artificial intelligence ethics, students' entrepreneurship

## Abstract

Students are the backbone of national development and progress, and should stand at the forefront of the era of innovation and entrepreneurship. Cultivating the entrepreneurship of college students is not only a response to the national call, but also a basic requirement for implementing quality education and promoting the comprehensive development of college students. To better study the entrepreneurship of college students, cultivate a group of newcomers of the era who have patriotic feelings, dare to innovate, hardworking and sustainable struggle, and solve the problem of college students' employment difficulties from the root, the cultivation mode of college students' entrepreneurship is studied. Firstly, the impact of artificial intelligence (AI) technology on social ethics is analyzed. Secondly, it analyzes the current situation of the cultivation of science and engineering college students' entrepreneurship from three aspects: Chinese traditional cultural thoughts influence the concept of career choice, enterprises emphasize utilitarianism, and colleges and universities attach importance to knowledge education and despise spiritual education. Finally, the data statistics on the cultivation of entrepreneurship of science and engineering college students are carried out in the form of questionnaires. The results demonstrate that among the students surveyed, 21.31% have a strong willingness to start their own business, and 72.84% have the idea of starting their own business, which means that most students still want to start a business through their own efforts, not blindly looking for jobs. Simultaneously, among many majors, 87.5% of students majoring in agriculture and medicine are better at finding new ways to solve problems, while the proportion of students majoring in literature and history is the lowest. It also indicates that most students believe that schools should add more seminar courses, internship courses, design courses, experimental courses, etc., and allow students to choose learning courses across colleges and majors, to cultivate college students' entrepreneurship. The proposed method provides some ideas for the application of AI technology in the cultivation of students' entrepreneurship.

## Introduction

Artificial Intelligence (AI) is one of the most popular technologies now, and has broad development prospects. The birth of this technology is like the emergence of steam engines, the application of power equipment and the Internet in life and industry, bringing new changes to human life and production, and bringing about great changes in all walks of life (Qian et al., 2018; Wang and Zhang, 2020). Nowadays, the use of AI technology has made human society develop rapidly, but rapid development has also brought many hidden dangers, such as ethical problems to human society. If this problem cannot be effectively solved, human beings cannot achieve long-term development (Jin et al., 2019). Considering that AI technology has become the mainstream technology of social development at this stage, the impact of AI on society is also multifaceted. Although it greatly drives a series of positive impacts such as driving the social economy and transforming people's lives. However, if there is a lack of management, there will be problems such as ethical anomie, moral anomie, safety out of control, and legal failure. Therefore, it is necessary for relevant entrepreneurs to strictly control when using AI technology to prevent the above problems from occurring (Gaies et al., 2022; Steininger, 2022). With the continuous expansion of the development and application of AI technology, the demand for a large number of industrial R&D talents, application development talents and practical skills talents will show a blowout trend. So, colleges need to adapt to the trend of the times, improve the talent training model, and actively promote the implementation of ideological and political teaching reform in AI majors, to cultivate high-quality future entrepreneurs with both morality and talent for social development. The purpose of the research is that entrepreneurship is the key driving force for stimulating market vitality, an inexhaustible source of promoting high-quality economic development, and an important factor in promoting the continuous development and growth of enterprises. In this new era of inheriting the past and ushering in the future, and carrying forward the excellent entrepreneurial spirit, it is of great significance for building a modern socialist country and realizing the Chinese Dream of the great rejuvenation of the Chinese nation. Faced with a new environment in the new era, it is necessary to continuously overcome many obstacles on the way forward. The construction of an innovative country is inseparable from college students in the new era. Meanwhile, college students in the new era also undertake the important mission of socialist construction. The spiritual heritage of college students has a profound impact on the development of the entire country. The relevant concepts of college students' entrepreneurship are defined. Combined with the professional knowledge of AI and the current background, the characteristics of the current college students' entrepreneurial spirit cultivation are clarified, emphasizing the importance and necessity of college students' entrepreneurship cultivation. On the premise of affirming the current research results of entrepreneurship cultivation, the problems existing in the cultivation of contemporary college students' entrepreneurship and the reasons for the problems are analyzed. Excellent various theoretical and practical achievements are absorbed, and the path of cultivating college students' entrepreneurship is improved, to cultivate a group of newcomers who have patriotic feelings, dare to innovate, hardworking and sustainable struggle.

Researchers have also carried out a lot of research work in related fields. Ayu and Nauly (2020) thought that entrepreneurship was crucial, which was the spiritual pillar of people's entrepreneurial process. The characteristics of entrepreneurs are analyzed, and a teaching model for cultivating young entrepreneurs who continue to engage in agriculture was found. The entrepreneurial factors affecting agricultural college students to continue to run family farms were analyzed. The results showed that risk, organizational skills, leadership, and effort influenced their decision to continue farming, while opportunities, patience, and other factors had little impact on their decision (Ayu and Nauly, 2020). Karayol and Taze (2020) found in the process of studying the entrepreneurship and employability of college students in the department of sports science: there were significant differences between teachers and students in the various dimensions of the employability scale and the entrepreneurial scale. This shows that there is a close relationship between the entrepreneurial ability and entrepreneurial spirit of college students (Karayol and Taze, 2020). Huang (2019) proposed and verified a new model of effectiveness measurement for entrepreneurship education, which measures the effectiveness of entrepreneurship education from three dimensions: entrepreneurial ability, entrepreneurial barriers and entrepreneurial intentions. This research takes 308 entrepreneurship education students from a key university in China as the research object, and explores the feedback patterns of three key variables. The results show that the improvement of participants' entrepreneurial ability, the reduction of entrepreneurial barriers and the change in entrepreneurial intentions reflect the effectiveness of college students' entrepreneurial education (Huang, 2019). Hagebakken et al. (2021) pointed out that entrepreneurship education is a means of creating economic activities and has vital implications for the development of rural areas. The research question is how education through entrepreneurship can meet the needs of entrepreneurial enterprises in rural settings. It found that participants believed that entrepreneurship education was very important to the local/regional entrepreneurial ecosystem, but participants mostly benefited during the course and to a lesser extent after the course. Sustainability played a role in attracting participants, but to a lesser extent constituted educational content and evaluation parameters (Hagebakken et al., 2021). Wang (2019) proposed that entrepreneurship was very significant and it made people interested in entrepreneurship. They analyzed the characteristics of entrepreneurs in an attempt to find the teaching model needed to cultivate young entrepreneurs who continued to work in agriculture. The results indicated that risk-taking, organization, leadership, and hard work variables significantly affected the decision of whether or not children should continue to work in agriculture (Wang, 2019). Combining domestic and foreign scholars' research on entrepreneurship, it can be concluded that: (1) Many scholars around the world have carried out research on entrepreneurship from multiple perspectives and put forward valuable viewpoints. Different scholars have different emphases on defining the concept of entrepreneurship. Socialism with Chinese characteristics has entered a new era, so it is a research point to explore entrepreneurship that is in line with the situation of Chinese entrepreneurs. (2) Compared with China, some foreign countries encouraged and cultivated entrepreneurial spirit earlier, so the path of foreign entrepreneurial spirit cultivation can be used for reference by China. However, the national conditions of each country are different, and advanced experience cannot be copied unrealistically. Therefore, combining the specific conditions of China and reasonably learning from foreign experience in cultivating entrepreneurship is the focus of the study. (3) Researchers in related fields in China have done a lot of research on the cultivation of entrepreneurship in other subjects. However, they have not proposed detailed optimization methods on the specific countermeasures for the cultivation of entrepreneurship of college students in the new era. This leaves room for research. By the current situation of the cultivation of entrepreneurial ability of foreign college students, it can be found that the cultivation of entrepreneurial ability of foreign college students started early and developed rapidly. The governments, societies, universities and families of various countries have given very positive encouragement and support, which is worth learning from. In view of foreign research on entrepreneurial ability cultivation and China's specific national conditions, China should take the cultivation of entrepreneurial talents as an important part of its economic and social development strategy. Rely on human capital to solve the problems of industrial structure adjustment, a transformation of scientific and technological achievements, job creation, and change in college students' employment concept. Looking at the domestic research on entrepreneurial ability and quality, it is believed that the research on entrepreneurial education in China has lasted for nearly 10 years, and the current research should not only stop at the qualitative aspects of the meaning, importance, and training methods of entrepreneurial education. Especially for colleges and universities, it is necessary to carry out qualitative and quantitative research methods for practical problems, summarize and analyze the cultivation of entrepreneurial ability and quality in colleges and universities. According to the specific situation of the school, the ways and countermeasures to carry out the cultivation of college students' entrepreneurial ability and quality are analyzed.

From the perspective of AI ethics, it mainly studies how colleges cultivate college students' entrepreneurship. By means of questionnaires, the entrepreneurship data of college students is analyzed, and the willingness of students to start a business and whether they have entrepreneurship are analyzed, to help colleges to better cultivate students' entrepreneurship and lay a spiritual foundation for them to become excellent entrepreneurs. The innovation point is: by establishing the content of the questionnaire with the elements of innovation and entrepreneurship as the core, the results of the survey can help colleges to understand the current situation of students more realistically, facilitate the colleges to formulate corresponding teaching plans, and better cultivate the entrepreneurship of students.

## Status Analysis and Questionnaire Design

### AI and Ethics

The University of Cambridge researched the topic “Whether your information has been stolen by AI algorithms” and found that AI algorithms do indeed steal users' personal information in the process of application. In terms of face recognition on mobile phones, which is the most used in daily life. When people easily master face recognition technology, personal privacy will be absolutely protected. When some criminals master face recognition technology, it will bring a great threat to people's property, privacy and even life. According to relevant data from People's Daily Online in 2018, as of the first half of 2018, there were 12,052 Internet fraud cases in China, with a total amount of 194.193 million yuan involved, which means that each person lost nearly 16,000 yuan, but Internet fraud still is constantly affecting people's lives. When people go to the bank to open related businesses and go to the supermarket to buy goods, they will use mobile phones. But meanwhile, personal information will be completely leaked to banks and merchants. There are still many ways of such information leakage, but there is no sound law to regulate it at this stage.

While AI brings development, it also brings challenges to the security of users' personal information, Internet fraud and privacy leaks. While endangering personal safety, it will also bring hidden dangers to national security. The problems faced by AI are not only ethical and moral issues, but also voice imitation, counterfeiting and deep forgery including face swapping, such as the lip-sync video of Donald Trump announcing nuclear war. The development goal of deep forgery technology is to make it easy for people with little technical ability to master its use. If so, it will pose a powerful threat to the world (Yan, 2018).

### The Current Situation of the Cultivation of Entrepreneurship of College Students

#### Traditional Cultural Thoughts of China Affect the Concept of Career Selection

Society is a large group for everyone to survive and develop. Because of the deep influence of traditional thinking, people are more willing to choose a stable way to live, which is also deeply rooted in people's hearts. And it traces back to 5,000 years of historical and cultural traditions in China, the golden mean is regarded as the principle of harmony, and is respected and followed by people. This understanding prompts the Chinese people to self-reflect in the face of various things, but it also makes people form a conservative way of thinking and personality traits (Eklund et al., 2020). Under the influence of traditional ideas, people's daily concepts are mostly stable and cautious, and they do not pursue a lifestyle of innovation and change, which leads to the whole society exploring the unknown and the atmosphere of innovation and entrepreneurship is not strong. Influenced by traditional Confucianism, most families adhere to the principle of “learning excellence makes an official,” and believe that the purpose of children's education is to seek stable and comfortable jobs such as civil servants, public institutions, and state-owned enterprises, etc. China has implemented a planned economy system for a long time, and the cultural background of encouraging students to innovate and start their own businesses is not deep enough, which constrains college students' innovative and entrepreneurial ideas. College students lack the courage to overthrow the past, and dare not try things that their predecessors have not done, which is not conducive to the cultivation of college students' entrepreneurship (Naqvi, 2021).

#### The Utilitarianism of Enterprises and Public Institutions

Enterprises and public institutions are a very important part of society, and the cultivation of college students' entrepreneurship requires not only the efforts of the school, but also the strong support of social enterprises. However, enterprises pursue more benefits and consider how to maximize the benefits obtained. They will not put their energy into the cultivation of college students, nor will they set up various entrepreneurial projects or fund internships for college students. Even if some enterprises are willing to give some support at the beginning, if they don't get benefits in the short term, they will become indifferent and finally stop giving economic support. This is because the enterprise is utilitarian, lacks a sense of social responsibility, and puts the maximization of interests first (Wu and Song, 2019). As a booster of economic development, it is understandable that enterprises pursue the maximization of benefits, but it is not worthy of vigorous advocacy to put utilitarianism first in the process of business, to magnify the value of money too much and ignore social interests (Lee and Raschke, 2020). Short-sighted, overemphasizing immediate interests and ignoring the potential utilization value that college students may bring, taking an indifferent attitude toward college students' creative ideas, innovative projects and innovative products, paying too much attention to timeliness, and impatience to invest too much human and financial resources to support high risks, high costs, but may be of extraordinary significance. The ultimate negative consequence of extraordinary projects is that these new technologies and products have died before birth and cannot really be put into practice to bring real economic and social value. Turning a blind eye to college students seeking support for innovation, and shutting out innovation and entrepreneurship achievements that may bring economic and social value to enterprises. College students finally gave up the road of innovation due to a lack of necessary social support (Hendiarto, 2021; Ridwan et al., 2021).

#### Colleges Pay More Attention to Cultivating Knowledge Than Cultivating the Spirit

In China, colleges devote more energy and time to imparting knowledge to students, placing too much emphasis on the cognitive ability of students. Teachers are educators and students are educated. The relationship between the two is one-way imparting and receiving. The standard to measure the quality of students' knowledge is grades, ignoring the cultivation of students' spirit of responsibility, honesty and trustworthiness, dedication, innovation and entrepreneurship, and active learning (Alhosseini et al., 2021). Colleges have not formed a joint force to cultivate entrepreneurship of college students, and have not stimulated the enthusiasm of college students to take the initiative to innovate and start businesses, causing some college students to have resistance to entrepreneurship, do not attach importance to the understanding of the spirit, but only to master knowledge and skills (Wu et al., 2018). This kind of cultivation method deviates from the direction of quality education advocated by the country. Meanwhile, under the influence of the social environment, to reduce the employment pressure of college students, college students can achieve short-term employment or entrepreneurship through a series of government assistance policies, so that the employment rate of graduates can be guaranteed. However, due to reasons such as the short time and excessive government support, college students are successful in entrepreneurship and employment in the early stage, but after reducing support in the later stage, innovation and entrepreneurship are weak and difficult to develop for a long time (Elliott et al., 2021; Peter et al., 2021). This kind of utilitarian educational method that ignores the cultivation of college students' own “spiritual” is not suitable for the general environment of social development. It is not advisable to follow objective laws and arbitrarily exert subjective initiative, which is inconsistent with the ultimate goal direction of the cultivation of entrepreneurship (Yohana et al., 2021).

### The Connotation of Entrepreneurship and the Model of Entrepreneurship Cultivation

It is well-known that the essence of entrepreneurship is the combination of opportunity awareness, innovation and risk-taking. Of course, entrepreneurship is not static, it will grow and change with the development of the times. The characteristics of entrepreneurship can be divided into three points, the first is acuity, that is, in order to pursue higher profits, entrepreneurs are generally the discoverers of market opportunities, and they have a very keen perception of changes in market information. The second is innovation. If an enterprise wants to be minimally affected by various business risks, it must understand and master the most core technologies in the industry. This puts forward certain requirements for entrepreneurs, requiring entrepreneurs to actively discover, use the latest inventions, use them in their own products and put them into the market. The third is responsibility. A successful entrepreneur must make appropriate decisions and take corresponding responsibilities in the ever-changing market environment. In the process of innovation and entrepreneurship, workers and managers need to find suitable opportunities according to changes in their surrounding environment and institutional culture, break through the original thinking framework, and achieve disruptive innovation. Therefore, in the process of carrying out innovation and entrepreneurship education (IEE), colleges and universities should cultivate students' entrepreneurship from the level of personal quality and social level. From the perspective of personal quality, different students' needs for success, risk preferences and the cultivation of innovative spirit make students more innovative, easy to break through the inherent thinking framework and behavior habits, and easy to adapt to the environment. From a social perspective, the development of entrepreneurship requires an atmosphere of innovation. Therefore, in the process of cultivating the social characteristics of entrepreneurship, the culture of tolerance for failure and innovation is conducive to the development of IEE.

The construction of a college entrepreneurial ecosystem can effectively carry out college students' entrepreneurial activities and promote social and economic development. In western countries, the entrepreneurship education ecosystem in colleges and universities is the main source of cultivating domestic entrepreneurial talents and promotes the development of the national economy. Therefore, it is necessary to establish a good IEE environment in colleges and universities, and form a benign entrepreneurial ecosystem, to improve the innovation and entrepreneurship ability of college students. The system needs to include multiple subsystems such as university, student, family, husband, business, etc. Through the interaction between the various systems, a complete IEE training system is formed. As the backbone of the IEE system, colleges and universities play an important role in the cultivation of entrepreneurial talents. As the recipients of IEE, college students need to learn relevant innovation and entrepreneurship concepts and carry out entrepreneurial activities in practice. The purpose of IEE in colleges and universities is to deal with the deficiencies in the cultivation of college students' innovative ability, entrepreneurial awareness and entrepreneurial ability in the traditional university education system. As a vital force to promote the development of society, innovation and entrepreneurship are critical factors to enhance China's international competitiveness. Therefore, in the process of implementing IEE, colleges and universities do not require college students to leave school to start a business, but to cultivate college students' innovative and entrepreneurial spirit, enhance their awareness of innovation and entrepreneurship. By learning the knowledge and skills of innovation and entrepreneurship, the risks that may arise in the process of entrepreneurship are prevented. Therefore, young entrepreneurs, as participants in the economic model of the new era, need to encourage and help college students with entrepreneurial and innovative abilities to boldly engage in innovative and entrepreneurial activities and provide spiritual guidance for entrepreneurial students. The current IEE model is relatively backward, the time and place are fixed, the teaching mode is single, the teacher's teaching and the students' passive learning is the main teaching mode. These have been unable to adapt to the development of the Internet and the entrepreneurial needs of students. The teaching content and teaching objectives of colleges and universities are out of touch with the actual needs. The course content lacks epochal, complex and applicable, and does not reflect the innovation of knowledge structure brought about by the development of science and technology. The education system of colleges and universities advocates focusing on the cultivation of academic talents, applied talents and entrepreneurial talents, but lacks corresponding teaching models, which cannot meet the needs of the development of vocational education and IEE in the new era.

### Design of Questionnaire

With the wider application of AI technology in society, how to ensure that the technology avoids ethical problems during the citation process requires enterprise managers to have high entrepreneurship. Therefore, when cultivating students in colleges, it is necessary to lay a good foundation of entrepreneurship for college students (Chen, 2021).

**Figure 1:**
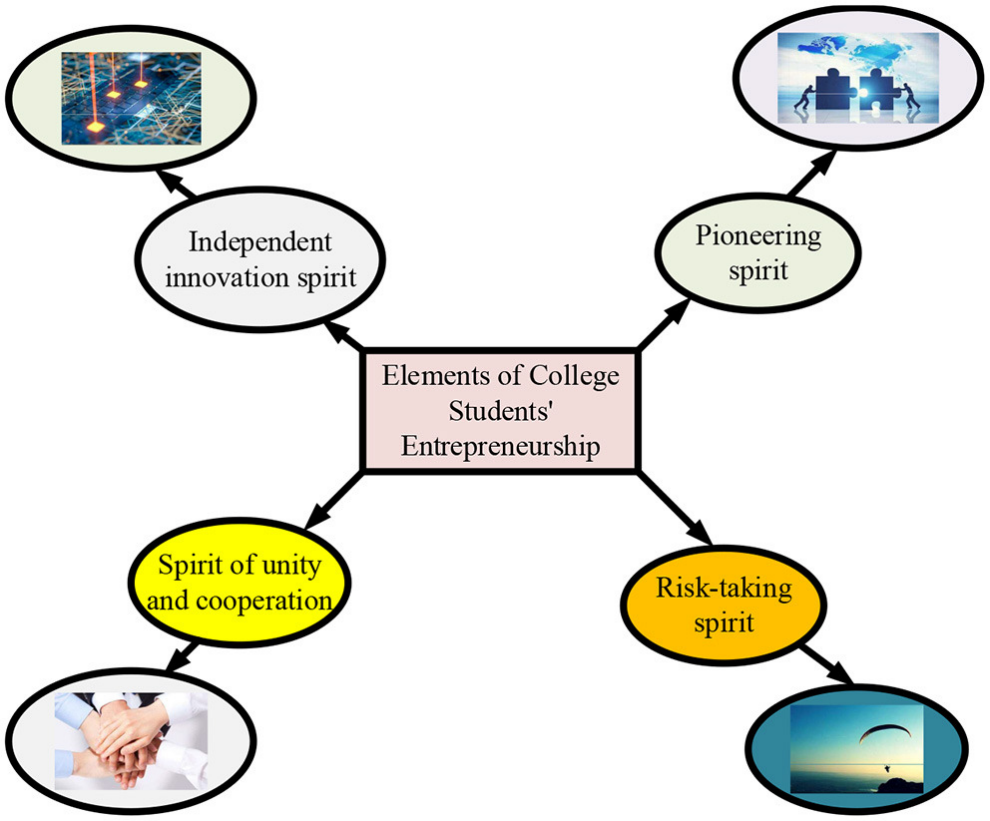
The elements of the cultivation of college students' entrepreneurship.

To more truly grasp the reality of college students' entrepreneurship and self-employment ability at this stage, a corresponding compensation plan is formulated according to their current limitations, to prevent ethical problems in society and help college students to better carry out self-employment (Alliance, 2018; Soegoto and Luckyardi, 2020; Kollmann et al., 2022). The elements of the cultivation of college students' entrepreneurship are shown in Figure 1.

It mainly adopts two methods: questionnaire and interview. The questionnaire contains two parts, one is basic information, and the other is college students' knowledge structure, entrepreneurial psychological quality, entrepreneurial ability and entrepreneurial personality. Through the questionnaire, the status quo of college students' innovation and entrepreneurship quality is recognized, and then the students who start their own businesses, entrepreneurship guidance teachers, and school leaders are deeply understood, and the existing problems affecting the cultivation of college students' entrepreneurial quality are analyzed and suggestions for improvement. It should be noted that because the freshmen do not have a good understanding of entrepreneurship and their awareness is weak, and colleges generally focus on innovation and entrepreneurship training for students in sophomores and above. So the research mainly focuses on college students who are sophomores, juniors and seniors.

The total number of questionnaires distributed this time is 300, and all questionnaires are recovered. The questionnaires with incomplete answers are eliminated, and there are 288 valid questionnaires, with an effective rate of 96%. In the later stage, through SPSS 25.0 software data processing, the current situation of college students' entrepreneurship is analyzed.

## Analysis of Results

### Sample Reliability and Validity Analysis

The mathematical expression of the alpha reliability coefficient can be written in Equation 1:


(1) $$\alpha =K\left(1-\sum \sigma_{i}^{2}/\sigma_{T^{2}}^{2}\right)/(K-1)$$

In Equation 1, *k* is the total number of questions (items) in the scale; $\sigma_{i}^{2}$is the intra-item variance of the score of the *i*th questions (items); $\sum \sigma_{i}^{2}$ is the sum of the variances of *k* item; $\sigma_{T^{2}}^{2}$ is the variance of the total score (sum of all questions).

The convergent validity and discriminant validity of the questionnaire are indicated in Table 1.

**Table 1:** The convergent validity and discriminant validity of the questionnaire.

**Variable**	**CR** **(Composite Reliability)**	**AVE** **(Average Variance Extract)**	**MSV** **(Maximm Shared Variance)**	**Initiative**	**Creativity**	**Insight and recognition**	**Opportunity utilization**	**Value-driven**	**Entrepreneurial personality**
Initiative	0.748	0.615	0.161	1.000					
Creativity	0.764	0.650	0.161	0.401***	1.000				
Insight and Recognition	0.781	0.628	0.142	0.306***	0.355***	1.000			
Opportunity utilization	0.704	0.578	0.114	0.214***	0.187***	0.338***	1.000		
Value-driven	0.716	0.586	0.070	0.265***	0.186***	0.102*	0.137**	1.000	
Entrepreneurial personality				0.562***	0.588***	0.673***	0.717***	0.755***	0.782***

^*^P < 0.05, ^**^P < 0.01, ^***^P < 0.001.

Table 1 refers that the AVE of the five factors of the questionnaire are all >0.5, and the CR > 0.7, which shows that it has good convergent validity. The correlation coefficient between each factor is smaller than the correlation between each factor and the total score, so the questionnaire can be considered to have good discriminant validity.

Table 2 indicates the criterion-related validity of the questionnaire.

**Table 2:** The criterion-related validity of the questionnaire.

**Variable**	**Initiative**	**Creativity**	**Insight and recognition**	**Opportunity utilization**	**Value-driven**	**Others**
Openness	0.162***	0.331***	0.235***	0.148**	0.110*	0.302***
Conscientiousness	0.123**	0.044	0.202**	0.106*	0.127**	0.176***
Extroversion	0.303***	0.352***	0.195***	0.171***	0.147**	0.343***
Pleasant	0.137**	0.018	0.083	−0.074	−0.046	0.058
Neuroticism	0.212***	0.174***	0.048	0.123**	0.066	0.163***
Active personality	0.658***	0.284***	0.217***	0.238***	0.172***	0.468***
Entrepreneurial self-efficacy	0.255***	0.186***	0.174*	0.276**	0.213***	0.324***

^*^P < 0.05, ^**^P < 0.01, ^***^P < 0.001.

Table 2 demonstrates that openness, conscientiousness, extroversion and neuroticism all have a low to moderate positive correlation with entrepreneurial personality. Active personality, entrepreneurial self-efficacy, entrepreneurial intention and entrepreneurial personality have a significant positive correlation.

The Cronbach's alpha coefficient of the questionnaire is 0.823, and the Cronbach's alpha coefficients of initiative, creativity, insight and recognition, opportunity utilization and value-driven are 0.742, 0.755, 0.776, 0.707, and 0.701, respectively. The test-retest reliability of the questionnaire is 0.774, and the test-retest reliability of initiative, creativity, insight and recognition, opportunity utilization and value-driven are 0.706, 0.732, 0.724, 0.665, and 0.717.

### Data Statistics of Questionnaires

#### Survey of Interests of Independent Innovation

**Figure 2:**
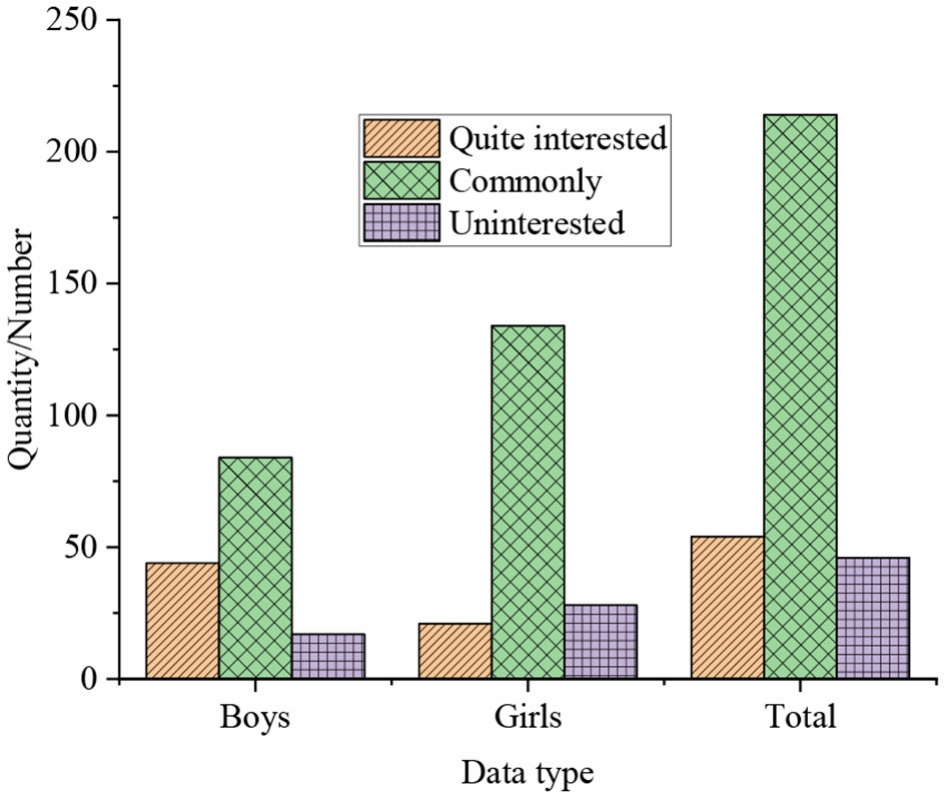
The results of the survey of college students' interest in independent innovation.

The results of the survey of college students' interest in independent innovation are shown in Figure 2.

In Figure 2, the statistics indicate that only 36% of the students show a high interest in a series of entrepreneurial plan competitions and innovative activities such as the challenge cup during their college years. 28% of the students are not interested in such activities, of which boys accounted for 12%, girls accounted for 16%. While the remaining students express moderate interest in such activities.

#### Survey of the Spirit of Independent Innovation

**Figure 3:**
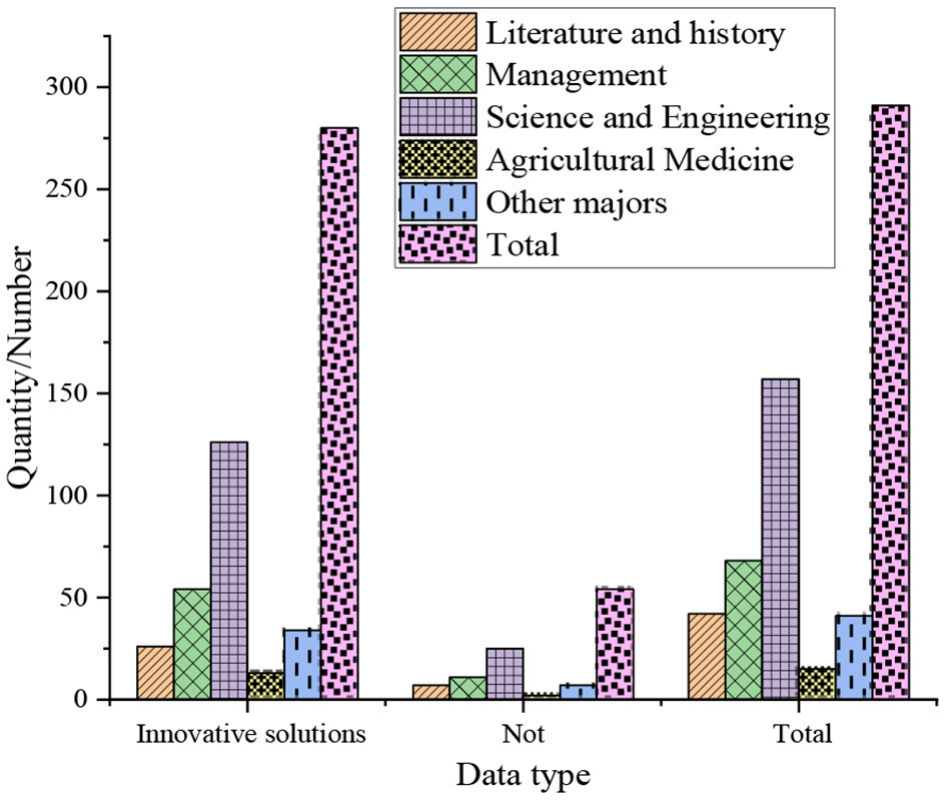
The results of the survey of college students' independent innovation spirit.

The results of the survey of college students' independent innovation spirit are shown in Figure 3.

The survey of the entrepreneurship of college students' independent innovation spirit shows that 87.5% of the students majoring in agriculture and medicine are better at finding new ways to solve problems. And 78.6% of students majoring in literature and history like to find new ways to solve problems through their own efforts, and this data is the lowest among all majors. This means that different majors have different entrepreneurship displayed by students.

#### Statistics on Self-Employment Willingness of College Students Under Different Genders

**Figure 4:**
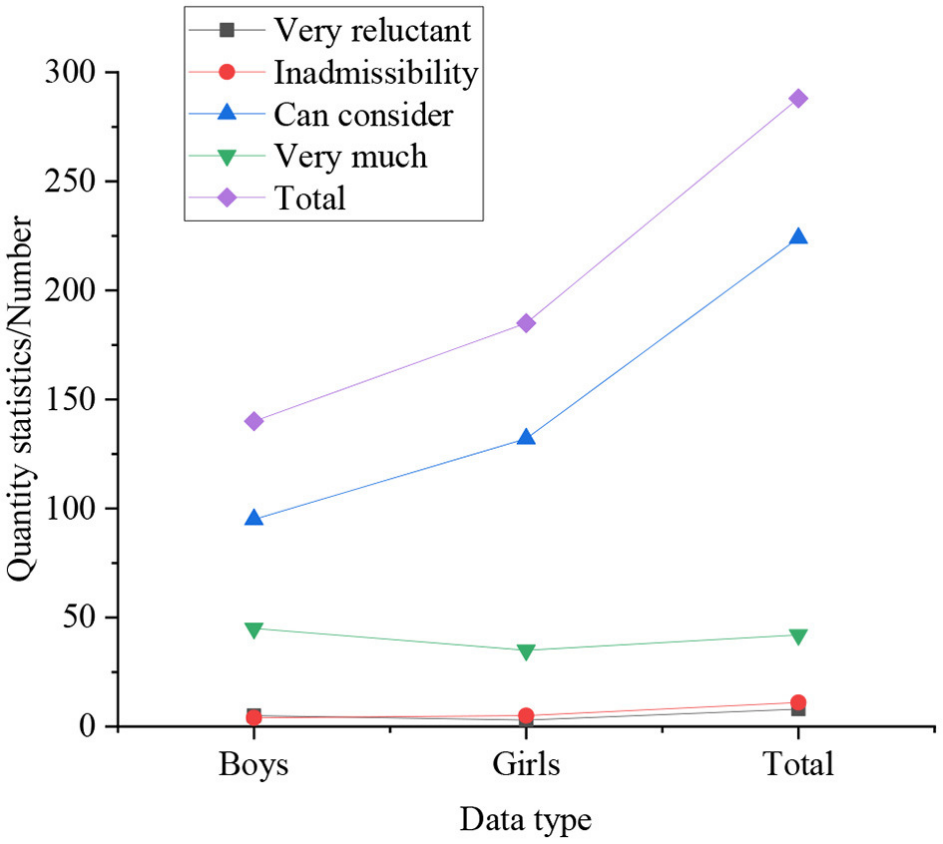
Statistical results of whether students of different genders have the willingness to start their own business.

**Figure 5:**
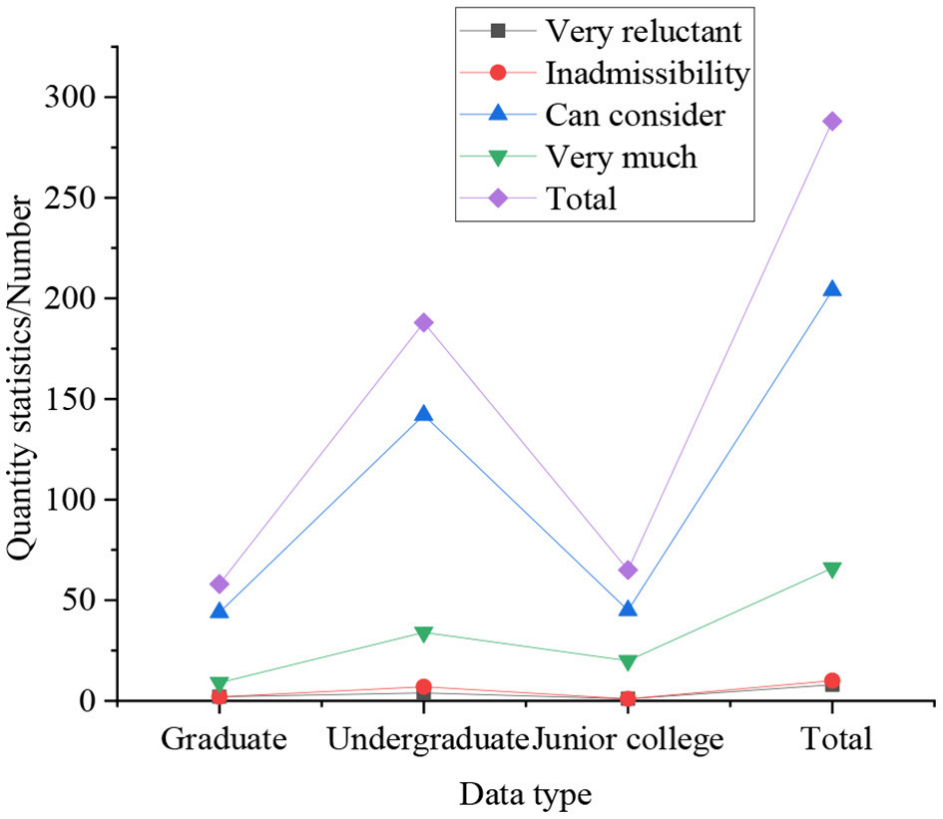
Statistical results of whether students of different educational backgrounds have the willingness to start their own business.

Figures 4, 5 are results of whether students of different genders and different educational backgrounds have the willingness to start their own business.

In Figures 4, 5, the statistical results express that 25% of boys have a strong willingness to start their own business, which is significantly higher than that of girls. Simultaneously, compared with other degrees, graduate students do not show a strong desire to start their own businesses, accounting for only 16%, followed by undergraduates, accounting for 19%, and college students with the strongest entrepreneurial desire, accounting for 30.77%. In the survey on self-employment willingness: the proportion of graduate students who are very reluctant is the highest, at 4.33%, followed by undergraduates, at 2.25%, and the lowest among students with college students, at only 1.92%. It indicates that with the improvement of academic qualifications, the intensity of students' willingness to start a business gradually decreases. It means that with the improvement of academic qualifications, the intensity of students' willingness to start a business gradually decreases. From the overall perspective of the respondents, 21.31% of the students have a strong willingness to start their own business, 72.84% of students have the idea of starting their own business, only 3.29% of the students have never thought about starting their own business, and the remaining students are very unwilling to start their own business. So most students still want to start a business through their own efforts, not just looking for a job.

#### Whether College Students Have a Persistent Entrepreneurship

**Figure 6:**
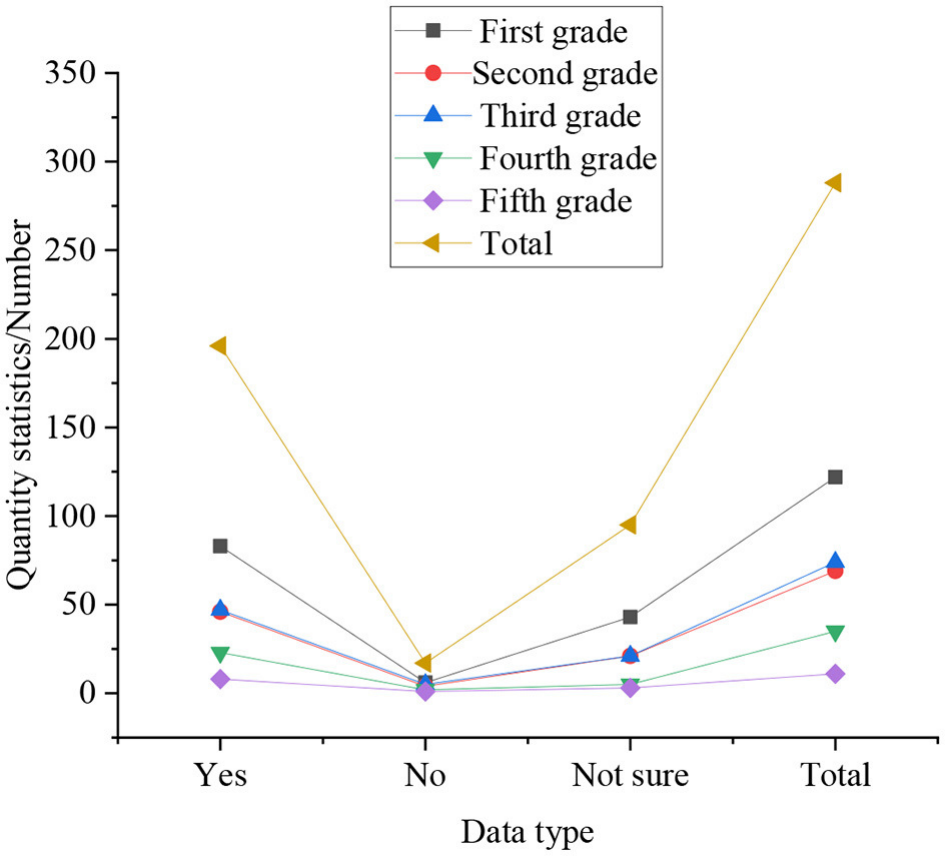
Statistical results of whether entrepreneurial college students have a persistent spirit.

Statistical results of whether college students have persistent entrepreneurship, as shown in Figure 6.

In Figure 6, the statistics represent that when contemporary college students encounter difficulties in entrepreneurship, life and study, the senior students are stronger than other grades, and can persevere in overcoming difficulties. The proportion is 76.64%, followed by the fifth-year students, accounting for 71.78%. Relatively speaking, the junior students do not show good entrepreneurship when facing various difficulties. Among them, juniors with entrepreneurship accounted for 31.26%. Sophomores accounted for 28.38%, and freshmen are 34.13%. In the statistics of entrepreneurship, 17% of seniors and 24% of fifth-year students are inconclusive. It means from this that the senior students have better entrepreneurial awareness and persistent entrepreneurship. from an overall statistics perspective: 64.2% of college students can complete tasks through their own efforts, showing persistent entrepreneurship, only 30.2% of students think that they are not sure whether they can overcome difficulties through their own efforts, but only 5.6% of students think they can't overcome difficulties.

#### Whether College Students of Entrepreneurship Can Cope With Emergencies

**Figure 7:**
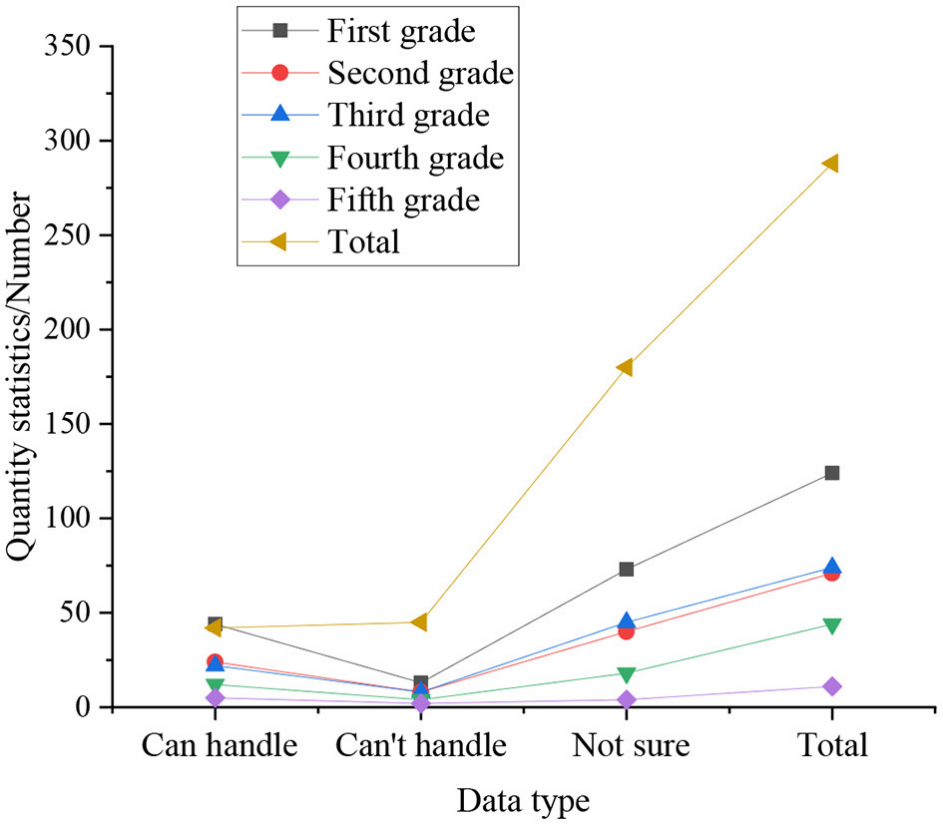
The statistics on whether entrepreneurial college students can cope with emergencies in the cultivation of entrepreneurship.

The statistics on whether entrepreneurial college students can cope with emergencies in the cultivation of entrepreneurship, as shown in Figure 7.

In Figure 7, the statistical results express that the fifth-year students are calmer in the face of emergencies in the process of self-employment, accounting for 43.18%, followed by the senior students, accounting for 37.23%, accounting for 37.23%. The smallest is the freshman, only 28.17%. In the statistics, among the students whose results are uncertain, the fifth-year students have the lowest proportion, which is 45.46%. Because after 5 years of study, the fifth-year students have a deeper understanding of themselves. Inversely, the proportion of the freshman is the highest in this aspect, accounting for 60.13%. Therefore, when college students encounter emergencies during self-employment process, the coping ability of the upper grades is much higher than that of the lower grades, because the upper grades are better than the lower grades in terms of knowledge reserve and psychological endurance. good. From the overall analysis, 31.78% of college students believe that they can cope well with emergencies in the process of entrepreneurship; 57.78% of college students are not sure whether they can deal with emergencies; 11.14% of college students feel that they can't deal with unexpected events in the process of starting a business.

### An Analysis of the Cultivating Courses of Entrepreneurship in Colleges

**Figure 8:**
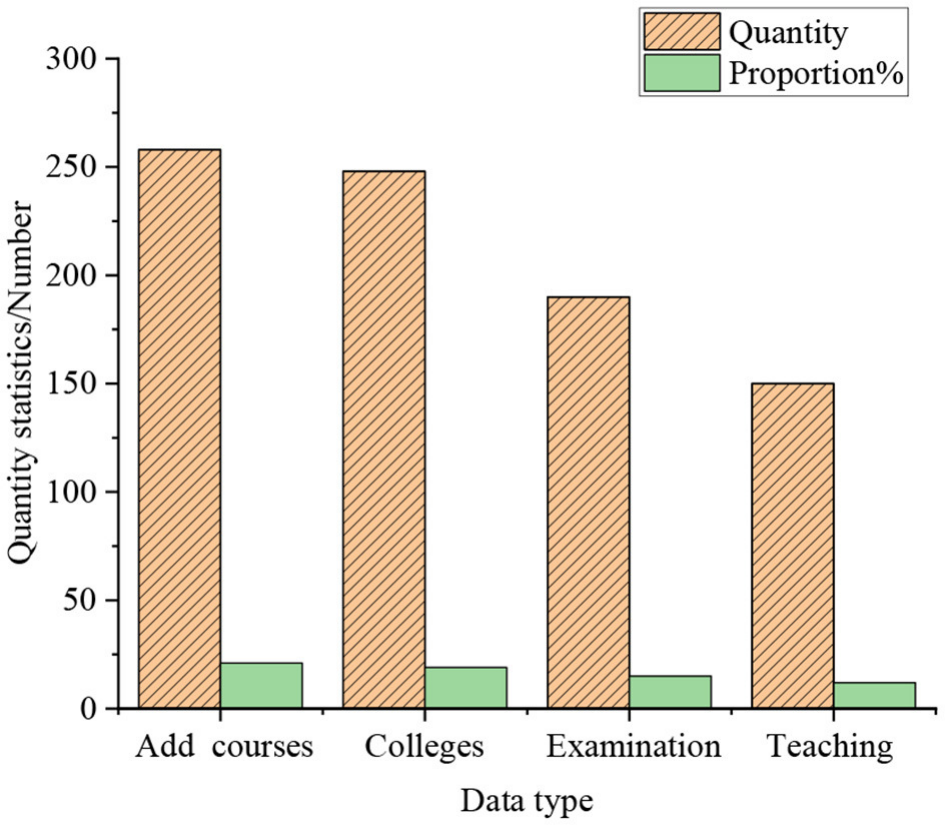
The statistics of the cultivating courses of entrepreneurship in colleges.

The statistics of the cultivating courses of entrepreneurship in colleges are shown in Figure 8.

**Figure 9:**
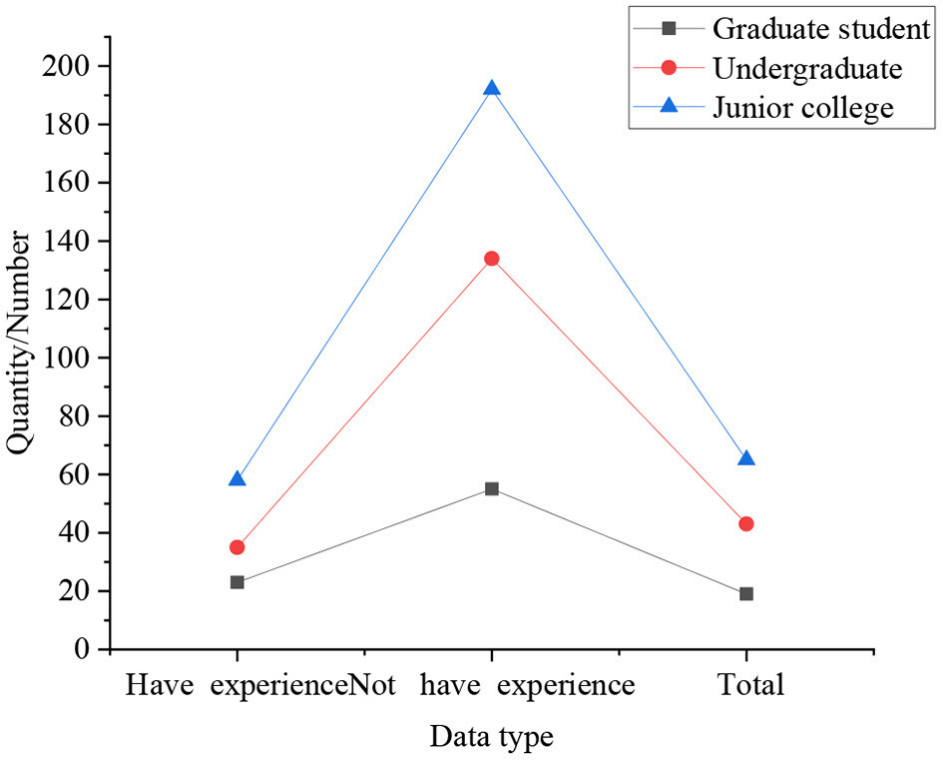
The results of the impact of the innovation and entrepreneurship competition on the entrepreneurship of college students.

Figure 8 indicates that 82.21% of college students think that schools should add more seminar courses, internship courses, design courses, experimental courses, etc., to help students take a job or start a business. 79.65% of the students believe that students could be allowed to choose courses of study across colleges and majors. 60.98% of the students consider that students could be examined by enriching their examination methods, rather than being limited to traditional paper-based examinations. 48.16% of students hold that the traditional cramming teaching model cannot cultivate students' entrepreneurship. From this, it means that at this stage, college students' demands for cultivating and teaching entrepreneurship are gradually becoming conscious. The analysis of the impact of the innovation and entrepreneurship competition on the entrepreneurship of college students is shown in Figure 9.

**Figure 10:**
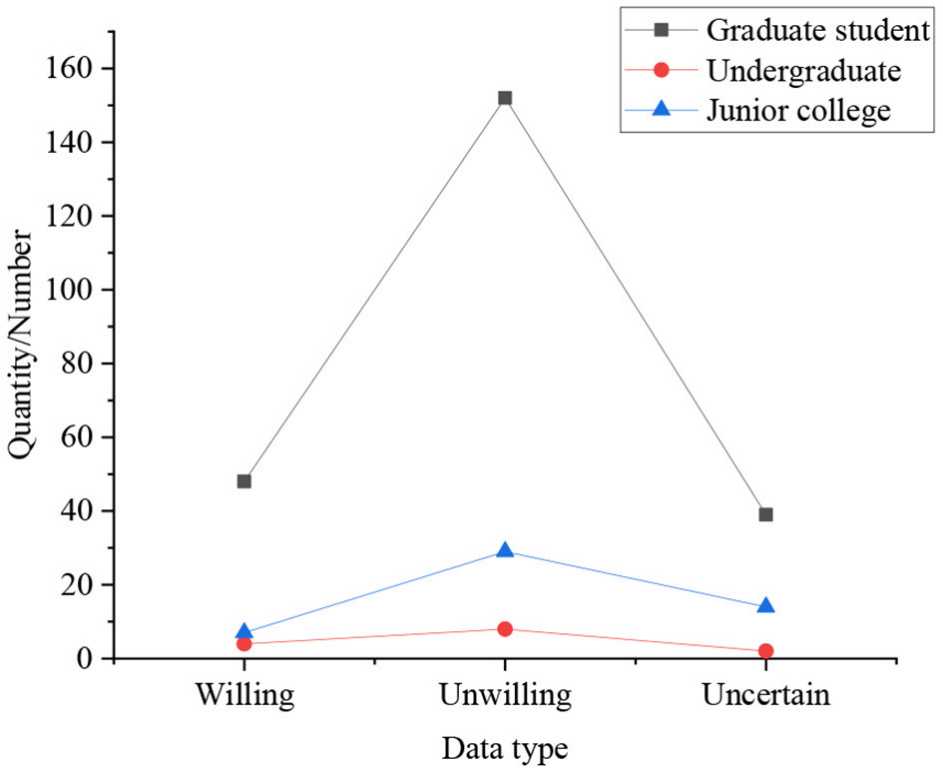
The impact of entrepreneurial views and ideas on college students during the entrepreneurial process.

Figure 9 expresses that 24.28% of students believe that participating in innovation and entrepreneurship competitions can help them more easily obtain external scientific help and achievements, and 20.19% of students think that it cannot help them obtain external scientific help and achievements. It shows from this that although most colleges in China have established “innovation and entrepreneurship incubators,” they have not played their role. Students are generally ineffective in obtaining scientific assistance and achievements, and they still need strong support from relevant departments. The impact of entrepreneurial views and ideas on college students during the entrepreneurial process is shown in Figure 10.

Combining the above experimental results, it manifests that among the students surveyed, 21.31% have a strong willingness to start their own business, and 72.84% have the idea of starting their own business. Simultaneously, among many majors, 87.5% of students majoring in agriculture and medicine are better at finding new ways to solve problems. 78.6% of students majoring in literature and history like to find new ways to solve problems through their own efforts, but this data is the lowest among all majors. It means that students show different entrepreneurship according to different majors. Moreover, the research also demonstrates that graduate students are more likely to accept entrepreneurial ideas and ideas than undergraduates and college students. Among them, about 83.12% of graduate students are willing to accept this view, while only 83.12% of undergraduates believe that this view is helpful for future entrepreneurship, and the proportion of college students is the lowest. Students consider that schools should add more seminar courses, internship courses, design courses, experimental courses, etc., and allow students to choose learning courses across colleges and majors, to cultivate college students' entrepreneurship and make them better able to take a job or start businesses.

### Discussion of Research Results

To sum up, it is not difficult to understand that at present, China's social and cultural atmosphere for encouraging college students to start their own businesses has not yet taken shape. As China's social and cultural atmosphere has always been deeply influenced by the traditional culture of Confucianism, it can be seen from this aspect that it lacks the cultural heritage of encouraging individual entrepreneurship. Therefore, people are generally content with the stable status quo, afraid of the unknown brought about by changes, accustomed to passive obedience and acceptance, and not good at actively engaging in innovation, creation and reform. Due to the influence of this concept, in the public's cognition, as a college student, you should study hard. Under the influence of this kind of psychology, most college students have developed the habit of “Regardless of external trivialities, be engrossed in books by the lofty and sagacious.” The common phenomenon is that they are content with the stable status quo, lack a sense of competition, have weak self-confidence, and cannot correctly recognize themselves. And they dare not to accept new challenges, dare not try risky activities, and dare not dare to take part in practice. The overall atmosphere of college students' self-employment has not yet been established; the entrepreneurial ability and quality have not been recognized by the society; the entrepreneurial practice has not been widely supported by enterprises and other institutions in the society. College students mainly learn knowledge in school, their innovation ability is not well cultivated, they have fewer opportunities to contact the society, lack social experience, and have a low ability to identify opportunities and integrate resources. Most of them stay at the theoretical level of books. It seriously affects the cultivation of college students' entrepreneurial ability and quality.

## Conclusion

College students' entrepreneurship has practical significance. 1. It is conducive to alleviating the current employment pressure of college students. And it is a diversion to the collective of college students. The entrepreneurial ability of college students is conducive to solving the problem of college students' employment difficulties. Entrepreneurial ability is the ability of self-survival and self-development in entrepreneurial practice activities. A college graduate with strong entrepreneurial ability will not only not increase the pressure of social employment, but will increase employment through self-employment. However, students with weak entrepreneurial ability need not worry, because ability can be acquired through learning and cultivation. 2. It is conducive to the realization of college students' own value. Through self-employment, college graduates can closely combine their interests with entrepreneurial projects, and do what they are most interested in, most willing to do, and what they think is most worthwhile to do. In today's society, they can show their abilities, maximize their talents, and get reasonable compensation. Although the current society encourages college students to start businesses from the perspective of resolving employment difficulties, the value (spiritual value and material value) generated by entrepreneurship is of great significance to individuals in society.

From the perspective of AI ethics, it mainly studies how colleges cultivate students' entrepreneurship. The results indicate that in the entrepreneurial activities of colleges, the proportion of boys participating is much higher than that of girls, which means that boys are more suitable for cultivating entrepreneurship. Meanwhile, among many majors, 87.5% of students majoring in agriculture and medicine are better at finding new ways to solve problems, while the proportion of students majoring in literature and history is the lowest. It refers that students show different entrepreneurship according to different majors. On the basis of previous research, a questionnaire survey is conducted. Combined with the questionnaire data, the current situation of entrepreneurship cultivation of college students in the new era is studied, the reasons for the existing problems are analyzed, and the cultivation countermeasures are put forward in a targeted manner. The contribution lies in digging out and sorting out the theoretical connotation of entrepreneurship after reading and analyzing a large number of domestic and foreign documents. From the aspects of the social atmosphere, cultivation system, cultivation mechanism, cultivation object, etc., this work analyzes the factors affecting the effectiveness of college students' entrepreneurship cultivation, and learns from the successful experience of foreign entrepreneurship cultivation. Based on the results of the questionnaire survey, the optimization measures for cultivation are proposed, which to a certain extent make up for the inadequacy of the previous cultivation of college students' entrepreneurship.

The research also has certain shortcomings. The designed questionnaire has relatively few questions, the dimensions of the questions are relatively single, and the distribution of the surveyed objects is relatively concentrated. This choice may have a certain impact on the results of the questionnaire. In addition, entrepreneurship is a dynamic and constantly changing system subject, which will change with the changes in regions, types of universities, and time. Therefore, the results of the questionnaire survey will change with time. This subject needs long-term follow-up research. The next step will be to optimize the content of the questionnaire, and simultaneously expand the number of survey objects and the scope of the survey.

At present, although the entrepreneurship education in colleges and universities has achieved fruitful results in the early stage, it is still necessary to closely follow the local development trend in the future development, complete the university transformation, cultivate more entrepreneurial talents that meet the needs of the region, and make due contributions to the development of entrepreneurial economy and the construction of entrepreneurial society in the region.

## Data Availability Statement

The raw data supporting the conclusions of this article will be made available by the authors, without undue reservation.

## Ethics Statement

The studies involving human participants were reviewed and approved by Hunan Normal University Ethics Committee. The patients/participants provided their written informed consent to participate in this study. Written informed consent was obtained from the individual(s) for the publication of any potentially identifiable images or data included in this article.

## Author Contributions

All authors listed have made a substantial, direct, and intellectual contribution to the work and approved it for publication.

## Conflict of Interest

The authors declare that the research was conducted in the absence of any commercial or financial relationships that could be construed as a potential conflict of interest.

## Publisher's Note

All claims expressed in this article are solely those of the authors and do not necessarily represent those of their affiliated organizations, or those of the publisher, the editors and the reviewers. Any product that may be evaluated in this article, or claim that may be made by its manufacturer, is not guaranteed or endorsed by the publisher.
